# Interns' Perceived Level of Proficiency After General Surgery Rotation: A Cross-Sectional Study From Saudi Arabia

**DOI:** 10.7759/cureus.57412

**Published:** 2024-04-01

**Authors:** Abdelkhalig Elhilu, Salman Ghazwani, Essa A Adawi, Siddig I Abdelwahab

**Affiliations:** 1 Surgery Department, Faculty of Medicine, Jazan University, Jazan, SAU; 2 Medical Research Centre, Jazan University, Jazan, SAU

**Keywords:** interns skills, internship challenges, surgical residency, surgical clerkship, internship training

## Abstract

Background: The role of interns during general surgical rotation is crucial in shaping their future careers as surgeons. Surgical rotation offers a unique opportunity to gain valuable hands-on experience in fast-paced and challenging environments. However, interns often face significant challenges in obtaining the necessary practical training to develop proficiency in surgical techniques. This article aims to analyze some aspects of the accumulated competency of interns during their general surgery rotation, focusing on the range of skills and knowledge gained, in addition to the challenges faced.

Subjects and methods: We conducted a cross-sectional study using an anonymous web-based self-assessment questionnaire. The target population of the study included all Jazan University medical interns enrolled in the academic year 2022-2023.

Results: Most participants showed low-to-average levels of proficiency in monitoring clinical evolution and treatment plans, ranging from fundamental awareness (n = 17, 17.5%) to working knowledge (n = 51, 52.6%), with only three participants (3.1%) reporting an expert level of proficiency. The same pattern was observed in the documentation of patient records (range: 7.2%, n = 7 for fundamental awareness to 42.3%, n = 41 for working knowledge). However, a significant proportion saw themselves as either proficient (n = 23, 23.7%) or experts (n = 15, 15.5%) in this aspect. Regarding bedside procedures, such as venipuncture, proctoscopy, nasogastric tube insertion, and urethral catheterization, the participants showed different proficiency levels, with the lowest in proctoscopy, where 66 (68.0%) of the participants reported only fundamental awareness. The results also revealed low perceived proficiency in performing surgical skin incisions, wound suturing, knot tying, application of surgical skin clips, and abscess drainage, with the lowest proficiency observed in the excision of superficial lumps as more than half of the participants reported only fundamental awareness (n = 51, 52.6%).

Conclusion: The results of this study indicate that documentation and monitoring of patient progress are the competencies mastered most by the majority of interns during their rotations in general surgery. However, the interns' overall level of proficiency in bedside procedures and basic surgical skills acquired during their rotation was low to average. Additionally, interns were dissatisfied with their training and the opportunities provided for them to actively engage in performing procedures in the operating room. This low proficiency is unrelated to pre-internship academic achievement, sex, or interest in future surgical careers. This suggests that efforts are needed to develop strategies to enhance interns' satisfaction and engagement, ultimately improving their overall experience during internships.

## Introduction

The transformation of medical students from the day they join a medical college to becoming well-established doctors passes through various stages. Wijnen-Meijer et al. identified six models or patterns that represent this transformation worldwide [1]. The most important phases of these models are medical schools, internships, and residency training. Internship serves as a vital experience that bridges the transition of medical students from undergraduate medical education to post-graduate residency training [2]. It is commonly defined as the period following undergraduate medical education and prior to becoming a fully licensed doctor. It consists of clinical work under supervision in accredited positions in hospital and primary care settings, typically lasting one to two years [3]. A rotation in general surgery is an integral part of internship and sub-internship preparatory courses in many countries worldwide [4,5].

However, several studies have raised concerns about trainees’ preparedness at different transitional stages of medical education in terms of their knowledge, abilities, and attitudes [5,6]. Traditional internship formats often lack organization, quality control, proper monitoring, and regular feedback when it comes to learning clinical skills [7].

In Saudi Arabia, an internship consists of a 12-month period of supervised training across various clinical disciplines. Interns spend two months in core rotations, which include general surgery, internal medicine, pediatrics, obstetrics, and gynecology. The remaining period is allocated to elective rotations in other specialties [8,9].

The role of interns during general surgery rotation is important in shaping their future careers as doctors. During general surgical rotation, interns are exposed to a variety of surgical procedures, ranging from routine to complex cases. By actively participating in the surgical team, interns acquire hands-on experience in preoperative evaluation, assisting in surgeries, managing postoperative care, performing bedside procedures, and patient follow-up [10]. They learn the fundamentals of surgical techniques, suturing, wound care, and the importance of maintaining a sterile environment in the operating room. General surgery rotations provide interns with ample opportunities to interact and collaborate with multidisciplinary teams, including attending surgeons, senior residents, anesthesiologists, nurses, and other healthcare professionals [9,11]. However, the extent of technical skill acquisition during general surgical rotations during internships remains largely unknown in Saudi Arabia. Self-reflection is a valuable tool in surgical intervention to assess proficiency in basic surgical skills. This involves critically analyzing their own performance during rotation and identifying areas for improvement. Mandel et al. showed that residents could rate themselves in task-specific performance and global skills with good reliability and validity [12]. This study aims to determine the perceived level of proficiency in technical skills gained by the participants during their rotations in general surgery and to identify the possible factors that may affect them.

## Materials and methods

We conducted this cross-sectional study using an anonymous web-based self-assessment questionnaire. The target population included all interns enrolled in the Jazan University Faculty of Medicine internship program during the academic year 2022-2023. All the participants in the study are graduates of the Faculty of Medicine at Jazan University. The Jazan University Faculty of Medicine internship program provides each intern with a logbook containing a methodical and detailed description of the skills that are supposed to be performed during each rotation. The target sample size was 105 participants. Of the total number of individuals who were approached to participate in the study, 97 responded to the questionnaire (92%). The interns’ perceived level of proficiency in different competencies was assessed using the National Institute of Health (NIH) proficiency scale. The NIH proficiency scale describes an individual’s proficiency level in a particular competency. It captures a range of ability levels and organizes them into five categories, ranging from “Fundamental Awareness” to “Expert" [13]. In addition to the demographic data, the questionnaire explored the self-perceived acquisition of a range of skills and competencies during their rotation in general surgery.

Data management and analysis

The study utilized data analysis, which was conducted using IBM SPSS Statistics (version 21.0). Data analysis involved several statistical techniques to examine the relationships between the variables. Descriptive statistics, including frequencies, percentages, mean values, and standard deviations, were used to summarize the data. The t-test and ANOVA were used to analyze the differences in the satisfaction scores. Binary logistic regression was then performed to investigate the relationships between the dependent variable (experience) and the independent variables (gender, age, future career aspirations, and satisfaction score). Adjusted odds ratios (AOR) were calculated to assess the strength and direction of these relationships, while p-values determined their statistical significance. The 95% confidence intervals provided a range within which the true AOR values were likely to fall, considering the effects of the other variables in the model.

Pilot study

A pilot study, consisting of 20 interns, was conducted to assess the reliability and suitability of the questionnaire. The results revealed high internal consistency for the experience and satisfaction scales, with Cronbach's alpha coefficients of 0.931 and 0.791, respectively. These findings indicate that the instruments used to measure the scales in this study are highly reliable. Consequently, the data collected from the pilot study, without any amendments to the data collection instruments, were included in the main study. This decision suggests that the researchers deemed the pilot study successful and opted to incorporate the interns' data into the main study. Overall, the pilot study confirmed the reliability and suitability of the questionnaire for evaluating interns' experiences and satisfaction.

## Results

The results of this study are summarized in Table 1. The table displays the frequency and percentage distribution of participants based on sex and age. Of the 97 participants, 54 (55.7%) were male, and 43 (44.3%) were female. Regarding age, 34 participants (35.1%) fell within the 20-24 age group, while 63 (64.9%) were aged ≥ 25 years. It also presents the frequency and percentage distribution of participants' intended future careers. Of the 97 participants, only 10 (10.3%) expressed an interest in pursuing a career in general surgery. Additionally, 18 participants (18.6%) indicated a desire to specialize in other surgical subspecialties, while 69 (71.1%) expressed interest in pursuing careers in other nonsurgical specialties.

**Table 1:** Distribution of participants by gender and age, career interest, and academic performance

Variables	Frequency	Percent	Satisfaction score	Test statistics (P-value)
Gender				
Male	54	55.7	13.96±3.88	1.57 (0.121)
Female	43	44.3	12.62±4.51	
Age				
20-24	34	35.1	14.12±4.63	1.29 (0.21)
25 and above	63	64.9	12.97±3.94	
Future career interests				
General surgery	10	10.3	10.40±1.67	2.90 (0.06)
Other surgical subspecialties	18	18.6	13.66±3.86	
Other non-surgical specialties	69	71.1	13.72±4.00	
Academic performance (CGPA)				
Less than 3.49	19	19.6	12.74±3.83	0.187 (0.905)
3.5-3.99	24	24.7	13.67±5.03	
4-4.49	39	40.2	13.46±4.06	
More than 4.5	15	15.5	13.47±3.93	
Total	97	100.0	13.37±4.20	

The results of the study regarding satisfaction scores and corresponding test statistics (p-values) are presented in Table 1. The table displays the satisfaction scores and the corresponding test statistics (p-values) for the different variables. For the gender variable, male participants had a satisfaction score of 13.96±3.88, while female participants had a slightly lower score of 12.62±4.51. The test statistic for this comparison was 1.57 (p-value = 0.121), indicating no statistically significant difference in satisfaction scores between the sexes. Regarding age, participants aged 20-24 had a higher satisfaction score of 14.12±4.63 compared to participants aged 25 and above, who had a score of 12.97±3.94. However, a test statistic of 1.29 (p-value of 0.21) suggested no significant difference in satisfaction scores between these age groups. In terms of future career aspirations, participants interested in general surgery had a lower satisfaction score of 10.40±1.67 compared to those interested in other surgical subspecialties (13.66±3.86) and other non-surgical specialties (13.72±4.00). The test statistic for the comparison between general surgery and other career aspirations was 2.90 (p-value = 0.06), indicating a borderline significant difference in the satisfaction scores. Overall, the total satisfaction score for all participants was 13.37±4.20.

The analysis of interns' perceived level of proficiency in different competencies, assessed using the NIH's proficiency scale, revealed interesting findings (Table 2). In competencies such as monitoring clinical evolution and treatment plans, 12.4% (n = 12) of interns reported fundamental awareness, 17.5% (n = 17) reported being a novice, 52.6% (n = 51) reported intermediate working knowledge, 14.4% (n = 14) reported advanced proficiency, and 3.1% (n = 3) reported an expert level of experience. Similarly, different levels of proficiency have been reported in areas such as the documentation of patient records, nasogastric tube insertion, urethral catheter insertion, scrubbing in the operating room, and various surgical procedures. The analysis also provided an overall total score with a mean of 4.816±15.9 (ranging from 20 to 100), representing the average level of proficiency across all competencies. These findings offer valuable insights into interns' self-perceived proficiency and skill development in various areas.

**Table 2:** The assessment of interns' perceived level of proficiency in different competencies was conducted using the National Institute of Health's (NIH) proficiency scale. Overall total score (mean±SD; minimum–maximum): 4.816±15.9 (20-100)

Level of experience	Fundamental awareness (basic knowledge)	Novice (limited experience)	Intermediate (working knowledge, practical application)	Advanced (applied theory and proficient)	Expert (recognized authority, can train others)
Competences and skills					
	N (%)				
Monitoring of clinical evolution of patients and treatment plan	12 (12.4)	17 (17.5)	51 (52.6)	14 (14.4)	3.0 (3.1)
Documentation of patient records	7 (7.2)	11 (11.3)	41 (42.3)	23 (23.7)	15 (15.5)
Nasogastric tube insertion	30 (30.9)	24 (24.7)	29 (29.9)	7 (7.2)	7 (7.2)
Urethral catheter insertion	26 (26.8)	26 (26.8)	26 (26.8)	13 (13.4)	6 (6.2)
Scrubbing in the operation room	12 (12.4)	5 (5.2)	28 (28.9)	23 (23.7)	29 (29.9)
Performing surgical draping	21 (21.6)	17 (17.5)	37 (38.1)	13 (13.4)	9 (9.3)
Performing surgical skin incisions	34 (35.1)	22 (22.7)	30 (30.9)	7 (7.2)	4 (4.1)
Performing knot tying	25 (25.8)	18 (18.6)	32 (33.0)	14 (14.4)	8 (8.2)
Performing wound suturing	21 (21.6)	26 (26.8)	31 (32.0)	12 (12.4)	7 (7.2)
Applying surgical skin clips	37 (38.1)	15 (15.5)	24 (24.7)	9 (9.3)	12 (12.4)
Performing wound debridement	13 (13.4)	18 (18.6)	33 (34.0)	18 (18.6)	15 (15.5)
Excision of superficial lumps	51 (52.6)	24 (24.7)	17 (17.5)	3 (3.1)	2 (2.1)
Chest tube insertion	53 (54.6)	23 (23.7)	16 (16.5)	1 (1.0)	4 (4.1)
Insertion of central venous line	58 (59.8)	19 (19.6)	18 (18.6)	0.0 (0.0)	2 (2.1)
Proctoscopy	66 (68.0)	18 (18.6)	10 (10.3)	1 (1.0)	2 (2.1)
Abscesses drainage	37 (38.1)	27 (27.8)	21 (21.6)	8 (8.2)	4 (4.1)
Venipuncture	49 (50.5)	10 (10.3)	20 (20.6)	12 (12.4)	6 (6.2)

**Figure 1:**
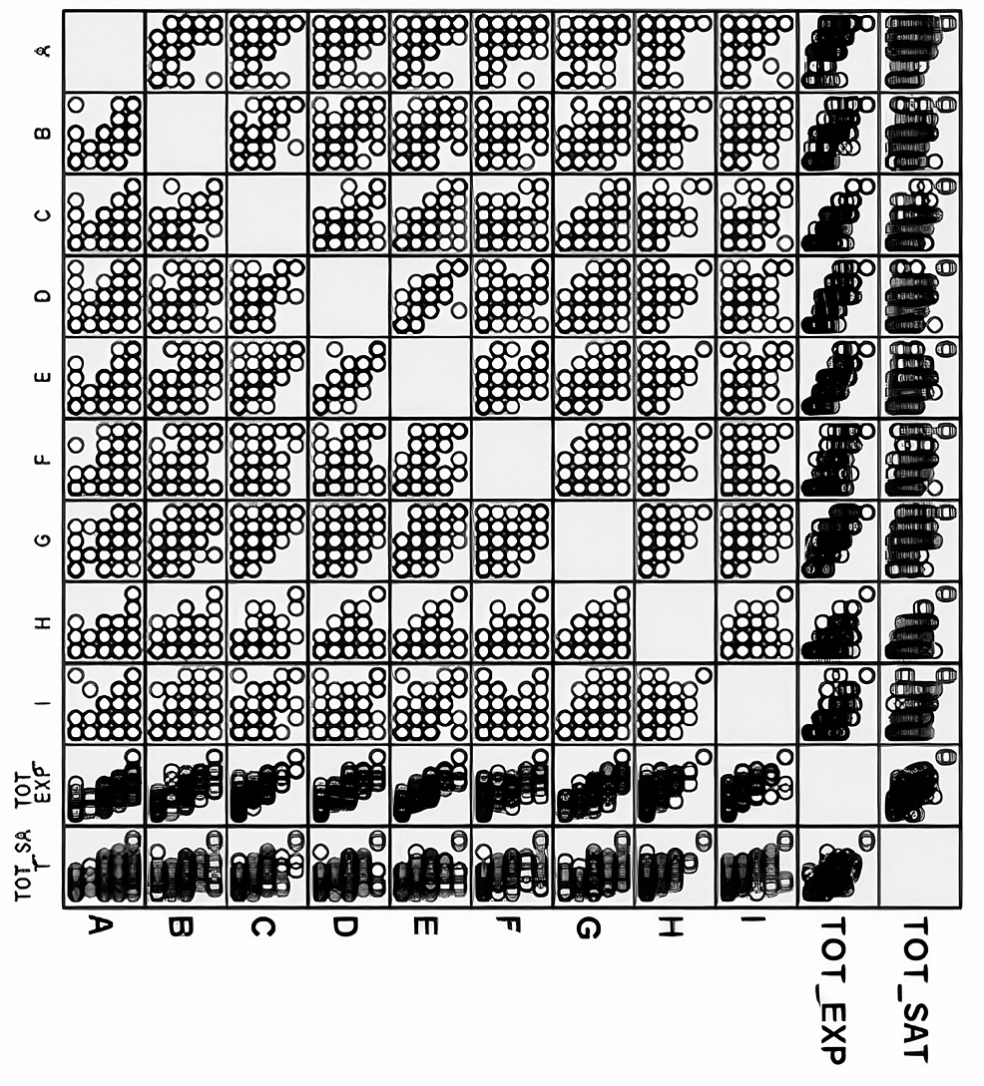
A correlogram showing the association between the total score for satisfaction related to opportunities given to practice surgical skills and competencies and the total score for proficiency items related to surgical skills and competencies A: SCRUBBING; B: SURGICAL DRAPING; C: SURGICAL_SKIN_INCISIONS; D: KNOT_TYING; E: WOUND_SUTURING; F: SURGICAL_SKIN_CLIPS; G: WOUND_DEBRIDEMENT; H: SUPERFICIAL_LUMPS; I: ABSCESSES; TOT_SAT: total score for satisfaction items related to opportunities to practice surgical skills and competencies; TOT_EXP: total score for experience items related to surgical skills and competencies. The correlation was performed using Spearman’s correlation due to the nature of the data and the abnormal distribution.

Figure 1 shows the correlogram for the association between the total score for satisfaction related to the opportunities given to the participants to practice surgical skills and competencies and the total score for the acquired experience or proficiency related to items of surgical skills and competencies. Each cell in the figure shows Spearman’s correlation coefficient between the two variables. For example, let us consider the correlation between satisfaction and “Scrubbing in the operation room": (A) The satisfaction score is 0.651, indicating a positive correlation between these two variables. The p-value was 0.005, suggesting that this correlation was statistically significant. The highest coefficient was observed between the satisfaction score and “Performing wound debridement.”

Table 3 presents the results of the binary logistic regression analysis, with proficiency (low and high) as the dependent variable and gender, age, future career, academic performance, and satisfaction scores as predictors. The results included AOR, p-values, and 95% confidence intervals (CI) for the predictors. The p-value for sex was 0.083, indicating no statistically significant association between sex and proficiency. The AOR for females compared to males was 0.328, with a 95% CI ranging from 0.110 to 1.146. The p-value for age was 0.079, suggesting that there was no significant association between age and proficiency. The AOR for participants aged 25 years and above compared to those aged 20-24 (reference category) was 0.328, with a 95% CI ranging from 0.093 to 1.137. In terms of future career aspirations, there was no significant association between proficiency and the choice of other nonsurgical specialties (p = 0.68). The AOR for participants interested in other surgical specialties compared with general surgery (reference category) was 6.58. However, there was a borderline significant association between experience and choice of other surgical subspecialties (p = 0.124). Interestingly, the satisfaction scores were significantly associated with proficiency (p = 0.00). For each unit increase in satisfaction score, the odds of having a high proficiency (compared to low proficiency) increased by a factor of 1.39. The 95% CI for the AOR ranges from 1.19 to 1.63. Overall, the results suggest that the satisfaction score is a significant predictor of proficiency, while gender, age, and future career aspirations do not show significant associations. The analysis revealed that students with a CGPA in the range of 3.5-3.99 have significantly lower odds (AOR: 0.12, 95% C.I.: 0.02-0.75) of having high proficiency compared to those with a CGPA less than 3.49. Similarly, students with a CGPA of 4-4.49 also exhibit lower odds (AOR: 0.32, 95% C.I.: 0.06-1.63) of high experience, although the association was not statistically significant (p = 0.17). Students with a CGPA higher than 4.5 have significantly lower odds (AOR: 0.08, 95% C.I.: 0.01-0.65) of high proficiency compared to the reference group. These findings suggest that academic performance, as measured by CGPA, is not uniformly consistent with the likelihood of having high proficiency.

**Table 3:** Results of Binary Logistic Regression Analysis AOR, adjusted odds ratio; CI, confidence interval; Ref, reference group.

Predictors	P –values	AOR	95% C.I.for AOR	
			Lower	Upper
Gender				
Male (Ref)				
Female	0.083	0.355	0.11	1.146
Age				
20-24 (Ref)				
25 and above	0.079	0.328	0.093	1.137
Future carrier				
General surgery (Ref)				
Other surgical subspecialty	0.124	6.58	0.595	72.79
Other non-surgical specialty	0.962	0.94	0.102	8.781
CGPA				
Less than 3.49 (Ref)				
3.5 - 3.99	0.02	0.12	0.02	0.75
4 - 4.49	0.17	0.32	0.06	1.63
More than 4.5	0.02	0.08	0.01	0.65
Satisfaction	< 0.001	1.39	1.19	1.63

## Discussion

A distinct set of abilities and knowledge is needed for internships rather than for academic classes. An internship places more of a focus on an intern's performance than their knowledge [14]. One of the key responsibilities of medical interns is to regularly document the progress of their patients [15]. This includes recording vital signs, laboratory results, medication administration, and any changes in the patient's condition. Documentation and monitoring of the clinical evolution of patients follows the SOAP format in hospitals where the participants of this study received their training [16]. Approximately one-quarter of the participants assessed themselves as proficient in patient-record documentation, while approximately 40% saw themselves as having a working grasp of the skill. A significant proportion see themselves as novices or have only fundamental awareness. Only a minority of the participants saw themselves as experts in the documentation of patient records. This result should raise some concern as inadequacy in this domain degrades the standard of patient documentation. However, these findings are not surprising considering the many studies that highlight the significant deficiencies in the quality of interns' patient records [17-19].

The proficiency of surgical interns in performing bedside procedures is paramount as it directly affects the quality of patient care. Interns must be adept at assessing, planning, and performing such procedures with precision and accuracy [20]. In terms of their ability to execute basic bedside procedures, most participants demonstrated limited competence in venipuncture, proctoscopy, nasogastric tube insertion, and urethral catheterization (fundamental awareness, novices, or just working knowledge). This pattern is even more evident in more invasive procedures such as chest tube insertion and central venous catheter placement. These gaps in the proficiency of interns in performing basic bedside procedures, which we detected in our study, are consistent with the results of other studies [21,22]. This could be caused by inadequate training in medical schools, a lack of opportunities to conduct these procedures, and subpar supervision during internship rotations [23]. Good supervisors, efficient supervision, adequate experiential learning opportunities, a supportive environment, a strong support system (including hospital staff, management, and academic opportunities), personal traits, and a manageable workload are all factors that provide good internship training [24].

Basic surgical skills such as wound closure, suturing, and dressing application are fundamental skills that interns are expected to master during internship training [25]. These skills form the foundation of complex surgical techniques and are essential for providing effective patient care. More than half of the participants saw themselves as proficient or experts in scrubbing, gowning, and gloving in the operating room. However, the majority did not perceive themselves as proficient in performing surgical draping. Additionally, they had low proficiency in performing surgical skin incisions, wound suturing, knot tying, application of surgical skin clips, excision of superficial lumps, and abscess drainage. These findings may suggest that they are not provided with sufficient opportunities to participate actively during operations. In response to an open question in our survey, one of the participants responded, "I think the major problem is that residents put the intern in a position to do only documentation rather than fairly distribute the cases to learn!". Another participant responded, "We need clinical teaching in the ward; clinic and OR, we need to get knowledge and information from the consultants and other doctors, we want also to give us the opportunity to work inside OR not just retraction and suction, we need someone who teach us how do suturing and let us practice". However, it is unclear in our study whether this is related to supervision and mentorship or due to a passive attitude from the participants toward attending the operating room and actively participating in surgical procedures. Magos et al. discovered that poor theater attendance is widespread among foundation-year-one doctors in the United Kingdom, regardless of rotation length, hospital type, or surgical specialty [26]. Moreover, the relatively short duration of the training period of our interns might have played a role, as our participants spent only eight weeks in general surgery rotation.

Other possible barriers to the development of technical skills proficiency, which has been highlighted by other studies, include fatigue from overwork, poor mentorship, failure to provide students with opportunities, time constraints, and inadequate skill training courses [27,28].

Our study found no significant association between the participants' future career interests and their overall proficiency scores. This could reflect a passive attitude towards being proficient in basic surgical skills, as more than 70% of the participants were not interested in general surgery or surgical subspecialties as prospective career preferences. However, the result is similar to the findings of Magos et al. in their study of foundation-year doctors [26].

The academic performance of the participants in medical school appears to be a poor predictor of performance during internships, as above-average students (CGPA: 3.5-3.99) have comparable odds to outstanding students (CGPA: > 4.5). This is consistent with the findings of other studies. Lee et al. reported conflicting outcomes in studies exploring the connections between undergraduate assessments and graduate performance by reviewing the literature [29]. Harfmann et al. concluded that residency performance cannot be predicted by a single medical student factor after a thorough analysis of the literature [30].

The correlation between the satisfaction of interns with the opportunities given to them and their skill proficiency acquisition during training is an important indicator of their achievement and learning experiences [31]. A higher skill proficiency level is often associated with a greater sense of achievement and fulfillment, leading to increased satisfaction and motivation. In our study, the positive correlation between the participants’ proficiency level in basic surgical skills and their satisfaction was clear, as shown in Figure 1. This is consistent with the findings of Luhoway et al., who identified failure to provide students with opportunities as one of the most common barriers to technical skill proficiency [27].

The gaps in technical skill proficiency during surgical rotations, which are highlighted by the results of this study, raise concerns regarding the status of interns' training during surgical rotations. Enhancing basic surgical skills is a continuous process that requires dedication, practice, and commitment to learning. The traditional apprenticeship model of surgical training, in which trainees learn through hands-on experience under the guidance of experienced surgeons, has long been the cornerstone of surgical education. However, in recent years, there has been growing concern about the sustainability of this model in the face of changing healthcare practices and increasing patient volumes [32,33]. Peyre et al. identified patient safety issues, work time constraints, shortage of opportunities in the operating room, and economic pressures as some of the reasons why programs are being pushed to supplement training with non-traditional methods of teaching surgical skills [34]. One of these non-traditional methods is the use of simulation in surgical training. Simulation-based courses within the medical school curriculum provide good learning opportunities for medical students and help improve their preparedness for clinical practice [28,35]. By creating a risk-free environment through simulation, students can gain practical knowledge that applies to their everyday patient interactions while boosting their self-confidence.

Good and effective supervision of interns is an important factor, which is vital to ensure that they receive the necessary training and guidance to become skilled and competent doctors. In recent years, there has been growing recognition of the need to enhance the supervision of surgical interventions to improve patient safety and overall quality of surgical care [31]. Effective supervisors provide their subordinates with responsibilities for patient care, opportunities to carry out procedures, opportunities to review patients, involvement in patient care, and direction and constructive feedback [36]. On the other hand, interns should actively seek opportunities to observe and assist in surgeries under the supervision of experienced members of the surgical team. By actively participating in these procedures, interns can gain valuable experience and become more proficient. For students to get the most out of their time in the operating room, Lyon identified three key domains: having a clear understanding of their learning objectives, having a measure of social competence that enables them to negotiate an active part of the team, and having the ability to cope with the environment of the theater and the emotional impact of surgery [10]. It is also important for surgical interns to seek feedback from senior surgeons and colleagues regarding their performance. Constructive criticism can help them identify areas for improvement and guide their efforts to enhance their skills [37]. Furthermore, we believe that interns should develop strong communication and teamwork skills. They need to communicate effectively with other members of the surgical team, including nurses, anesthesiologists, and other surgeons.

Limitations

This study had some limitations. Data were collected from a single academic institution, which limits the generalizability of the results. Future research should involve larger and more diverse samples to enhance external validity. Furthermore, longitudinal studies can explore the dynamic nature of career experience and investigate its influencing factors over time.

## Conclusions

The results of this study indicate that documentation and monitoring of patient progress are the competencies mastered most by the majority of interns during their rotation in general surgery. However, the interns' overall level of competency in bedside procedures and basic surgical skills acquired during their rotation was low to average. Additionally, interns were dissatisfied with their training and the opportunities provided for them to actively engage in performing procedures in the operating room. This low proficiency is unrelated to pre-internship academic achievement, sex, or interest in future surgical careers. This suggests that efforts are needed to develop strategies to enhance interns' satisfaction and engagement, ultimately improving their overall experience during internships.
